# Epidemic preparedness: Prenatal Zika virus screening during the next epidemic

**DOI:** 10.1136/bmjgh-2021-005332

**Published:** 2021-06-11

**Authors:** Luxi Qiao, Celina M Turchi Martelli, Amber I Raja, Nuria Sanchez Clemente, Thalia Velho Barreto de Araùjo, Ricardo Arraes de Alencar Ximenes, Demócrito de Barros Miranda-Filho, Anna Ramond, Elizabeth B Brickley

**Affiliations:** 1 Health Equity Action Lab, Department of Infectious Disease Epidemiology, London School of Hygiene & Tropical Medicine, London, UK; 2 School of Medicine, Washington University in St Louis, St Louis, Missouri, USA; 3 Instituto Aggeu Magalhães, Fundação Oswaldo Cruz, Recife, Pernambuco, Brasil; 4 Departamento de Medicina Social, Universidade Federal de Pernambuco, Recife, Pernambuco, Brasil; 5 Departamento de Medicina Tropical, Universidade Federal de Pernambuco, Recife, Pernambuco, Brasil; 6 Departamento de Medicina Interna, Universidade de Pernambuco, Recife, Pernambuco, Brasil

**Keywords:** Screening, Child health, Maternal health, Arboviruses, Public Health, Zika virus

## Abstract

Zika virus (ZIKV) is a vectorborne infectious agent of global public health significance due to its potential to cause severe teratogenic outcomes. The question of whether health systems should consider adopting screening programmes for ZIKV infections during pregnancy warrants consideration. In this analysis, we apply the Wilson-Jungner framework to appraise the potential utility of a prenatal ZIKV screening programme, outline potential screening strategies within the case-finding pathway, and consider other epidemiological factors that may influence the planning of such a screening programme. Our evaluation of a potential prenatal ZIKV screening programme highlights factors affirming its usefulness, including the importance of Congenital Zika Syndrome as a public health problem and the existence of analogous congenital prenatal screening programmes for STORCH agents (syphilis, toxoplasmosis, others (eg, human immunodeficiency virus, varicella-zoster virus, parvovirus B19), rubella, cytomegalovirus, and herpes simplex virus). However, our assessment also reveals key barriers to implementation, such as the need for more accurate diagnostic tests, effective antiviral treatments, increased social service capacity, and surveillance. Given that the reemergence of ZIKV is likely, we provide a guiding framework for policymakers and public health leaders that can be further elaborated and adapted to different contexts in order to reduce the burden of adverse ZIKV-related birth outcomes during future outbreaks.

**Summary box**

- While it is well established that Zika virus (ZIKV) infections during pregnancy can have deleterious impacts on the health and well-being of congenitally infected offspring and their families, the question of whether prenatal screening for ZIKV may be warranted during epidemics remains open.
- Our analysis of a potential prenatal ZIKV screening programme using the Wilson-Jungner framework highlights factors affirming the utility of screening, such as the importance of Congenital Zika Syndrome as a public health problem, a growing understanding of the natural history of the disease, and the likely acceptability of a programme.
- However, our assessment also reveals key barriers to implementation, especially related to diagnostics, antiviral treatments, social service capacity, and surveillance.
- Adopting prenatal screening for ZIKV during an epidemic would enable pregnant persons to make more informed decisions about their pregnancy and facilitate the early identification of exposed newborns for specialised follow-up care, which may be of particular importance for children who present asymptomatically at birth but develop ZIKV-related sequelae in later childhood.
- Nevertheless, to be most impactful, prenatal screening programmes require cheaper and more precise ZIKV screening tests, as well as continued investment in the development of efficacious and safe therapeutics.

## Introduction

Screening programmes are a core public health service and can be a valuable tool in improving a population’s health outcomes.^1^ The purpose of screening is to identify individuals in a ‘healthy’ population who are at higher risk of a specific health condition, in order to provide early treatment and/or intervention to high-risk individuals and thereby reduce the incidence of and/or mortality due to the condition in the full population.^1^ Prenatal screening programmes for congenital conditions, including those caused by infectious diseases like Zika virus (ZIKV), aim for the early detection of risk factors for fetal anomalies in order to enable expectant parents to make informed choices about their pregnancy and aftercare.^1^

Similar to other STORCH (syphilis, toxoplasmosis, others [eg, HIV, varicella-zoster virus, parvovirus B19], rubella, cytomegalovirus and herpes simplex virus) agents, ZIKV can be vertically transmitted across the placenta during pregnancy with potentially deleterious consequences for fetal development. Unlike other STORCH agents, ZIKV is vectorborne, and its transmission by competent *Aedes* spp. mosquitoes^2^ can facilitate explosive outbreaks with spatial and temporal clustering of neonates born with Congenital Zika Syndrome (CZS).^3–5^ As of July 2019, a total of 87 countries and territories across Africa, the Americas, South-East Asia and the Western Pacific reported evidence of autochthonous mosquitoborne transmission of ZIKV, while an additional 61 countries and territories have demonstrated evidence of established *Aedes* spp. vectors without yet having documented ZIKV transmission.^6^

Given the severity of outcomes associated with congenital ZIKV infection and the threat of ZIKV reemergence in areas with prior outbreaks or emergence in new settings,^7^ the adoption of a screening programme for ZIKV infections during pregnancy warrants consideration. Here, we draw on the Wilson and Jungner evaluative framework^8^ to analyse the feasibility and appropriateness of a prenatal screening programme during a ZIKV epidemic (summarised in table 1).

**Table 1:** Summary of points supporting and not supporting each criterion of the Wilson and Jungner screening framework, with additional recommendations for futher research and/or public health actions

Criteria	Points supporting the criterion	Points not supporting the criterion	Recommendations for further public health action(s) and/or research
1. The condition sought should be an important health problem	ZIKV infection during pregnancy may lead to cases of CZS, which can pose a significant burden at the individual, family, and societal level.	ZIKV infection during pregnancy occurs at a lower frequency between outbreaks in areas with arbovirus circulation.	Public health surveillance for ZIKV epidemics to inform the timing and type of prenatal ZIKV screening programme. Research on the long-term health, social and economic impacts of CZS on affected children, families, and communities.
2. There should be a suitable test or examination	Validated and acceptable molecular and serological tests for detecting ZIKV infections during pregnancy are currently available.	Molecular tests have narrow windows of detection. Serological tests are subject to immunological cross-reactivity with other flaviviruses.	Combining highly specific molecular tests and highly sensitive serological tests to allow for more accurate identification of ZIKV infections. Research to develop diagnostic tests with enhanced sensitivity and specificity. Research to evaluate performance of tests in settings with endemic flavivirus circulation.
3. The natural history of the condition, including development from latent to declared disease, should be adequately understood	Children with CZS who survive infancy are expected to have a prognosis comparable with children who have other conditions associated with microcephaly, epilepsy, and intellectual disability.	Significant heterogeneity remains in the risk estimates of adverse outcomes associated with prenatal ZIKV exposure, in part due to inconsistencies in the range of outcomes assessed. The prognosis of children with prenatal ZIKV exposure remains uncertain beyond 5 years of age.	Expansion of infrastructure and governance policies for sustained data sharing between Zika cohort studies. Research using individual participant data meta-analyses of pregancy and paediatric cohorts and standardised outcomes to improve the precision of risk estimates. Research using paediatric cohort studies and linked electronic health records to follow up children with prenatal ZIKV exposure born with or without apparent manifestations of CZS.
4. There should be a recognisable latent or early symptomatic stage	Sensitive and specific laboratory diagnostic assays have the potential to detect maternal ZIKV infections before the onset of severe fetal anomalies.	Antivirals for impeding vertical transmission of ZIKV are not currently available. Prenatal and postnatal testing to identify offspring who may be ZIKV-exposed but uninfected remains limited. A large portion of ZIKV infections are asymptomatic.	Public health efforts to improve antenatal care attendance. Monitoring of children with prenatal ZIKV exposure who present asymptomatically at birth for developmental delays and late-onset sequelae of congenital infections. Research to develop antiviral treatments to block vertical transmission. Research to improve the safety and effectiveness of testing for offspring infection status (eg, via amniocentesis or neonatal serum/urine testing).
5. The test should be acceptable to the population	Non-invasive prenatal screening tests for STORCH agents are generally well accepted.	There is limited research on the attitudes towards prenatal screening tests for ZIKV.	Public health education and communication on risks of prenatal ZIKV infections and screening methods. Research on attitudes of expectant families in ZIKV-endemic settings.
6. There should be an agreed policy on whom to treat as patients	Both the pregnant person and offspring would be considered as patients due to the potential for vertical transmission of ZIKV infection during pregnancy.	Local policies may differ with regards to the rights of pregnant persons and their offspring (eg, with respect to pregnancy terminations).	Prenatal counselling following positive screening test result. Postnatal clinical and social support for children with prenatal exposure to ZIKV and their families. Research to evaluate indirect effects of CZS on caregivers’ mental health, social support, and lived experiences.
7. There should be an accepted treatment for patients with recognised disease	Anticipatory guidance to caregivers and early referrals to appropriate specialists and early intervention programmes for the affected child and family is well accepted.	There are no available vaccines or antiviral treatments. Access to unrestricted legal abortion remains rare in many settings where ZIKV is endemic.	Expanded access to specialised treatment programmes for children with CZS. Research to develop vaccines to prevent ZIKV infection during pregnancy. Research to develop antiviral treatments to prevent vertical transmission.
8. Facilities for diagnosis and treatment should be available	Diagnostics and expertise developed during the last ZIKV epidemic exist.	Utilisation of existing ZIKV diagnostic assays require significant resources. Follow-up care for CZS patients and their families requires long-term financial commitment from health systems.	Public health efforts to improve accessibility and affordability of tests. Research to develop rapid, low-cost, point-of-care testing. Research into effectiveness of interventions in clinically relevant subgroups to inform targeted treatment.
9. The cost of case finding should be economically balanced in relation to possible expenditure on medical care as a whole	Screening is likely to be most cost-effective during an outbreak situation.	In non-outbreak situations, screening may not be cost-effective, as screening costs could be more than the preventable costs.	Enhancing capacity to rapidly integrate ZIKV screening into existing prenatal testing platforms during outbreak situations. Research to develop multiplex prenatal screening assays, including ZIKV testing.
10. Case finding should be a continuing process and not as a once-and-for-all project	Ultrasound may be used with or in lieu of laboratory testing to detect cases of CZS. Routine paediatric evaluations and developmental screening may be used to detect CZS-related anomalies at a later stage.	Maternal ZIKV infections may be asymptomatic, and ZIKV-exposed infants may present without typical CZS features.	Increased monitoring of pregnancies during an outbreak situation. Continued monitoring of symptomatic and asymptomatic children with prenatal ZIKV exposure for developmental delays and late-onset sequelae of congenital infections. Research evaluating paired mother-offspring testing to detect congenital infections postnatally.

CZS, Congenital Zika Syndrome; STORCH, (syphilis, toxoplasmosis, others (eg, human immunodeficiency virus, varicella-zoster virus, parvovirus B19), rubella, cytomegalovirus, and herpes simplex virus); ZIKV, Zika virus.

## Considerations for assessing a prenatal screening programme for ZIKV infection

### 1. The condition sought should be an important health problem

Whereas maternal ZIKV infections are generally mild and self-limiting, a subset of congenital ZIKV infections may disrupt fetal development and result in structural malformations and functional neurodevelopmental impairments, which are collectively recognised as CZS.^9–11^ Although there is a wide spectrum of CZS severity, children with more severe cases of CZS are likely to have long-term needs for caregiving, medication and specialised support, which can incur substantial social and economic costs for affected individuals, their families and wider society.^12^ Additionally, evidence from a nationwide retrospective study linking routinely collected data in Brazil from 2015 to 2017 reported a case fatality rate of 9.4% (95% CI: 8.4% to 10.6%) among children with confirmed CZS diagnoses, with more than 90% of deaths occurring in infancy.^13^

### 2. There should be a suitable test or examination

ZIKV infections during pregnancy are primarily identified using nucleic acid amplification and serological tests. Nucleic acid amplification tests detect the presence of ZIKV RNA (ie, indicating acute infection) by reverse transcription-polymerase chain reaction (RT-PCR) in maternal whole blood, serum, plasma, urine and amniotic fluid in patients presenting, in general, <7 days from onset of symptoms, and serological tests detect binding and neutralising antibodies (ie, indicating acute or prior infection) against specific ZIKV antigens (eg, NS1) in whole blood, serum and plasma in patients presenting ≥7 days from symptom onset.^14^ Ideally, a combination of the high specificity of molecular testing and the high sensitivity of serological testing for ZIKV would potentially allow for the most accurate identification of infections.^15^ While individual assay performances are highly variable and can depend on the type and timing of sample collected and, in the case of serological assays, the immunological cross-reactivity with other flaviviruses (eg, dengue virus (DENV)), the overall performances of ZIKV-specific assays are largely similar to the performances of routine diagnostic tests for other STORCH agents during pregnancy.^16^ Integrating ZIKV into the STORCH screening paradigm has the potential to improve standardisation and the differential diagnosis of other congenital infections.

### 3. The natural history of the condition, including development from latent to declared disease, should be adequately understood

The generally mild clinical presentation and the co-circulation of other immunologically cross-reactive flaviviruses in ZIKV-endemic areas has made establishing a case definition for ZIKV infection in pregnant persons challenging.^15^ A series of population-based prospective cohort studies (table 2) provide evidence that among pregnant individuals with laboratory-confirmed ZIKV, the risk of congenital ZIKV infection ranged between 5% and 35%, and the risk of adverse pregnancy and birth outcomes ranged between 2% and 46%,^17–25 66^ with the high variability in adverse risk estimates due, in part, to the differing lengths of follow-up and outcome definitions used between studies. Children with CZS who survive infancy are expected to have a prognosis comparable with that of others who have conditions associated with microcephaly, epilepsy, and intellectual disability.^26^

**Table 2:** Prospective cohort studies studying absolute risks of adverse outcomes following maternal ZIKV infection

Location	Enrolment period	Enrolment requirement	% with laboratory confirmed maternal infection (n/N)	Laboratory test for maternal infection	% with laboratory confirmed congenital infection (n/N)	Laboratory test for congenital infection	% of absolute risk for adverse outcomes* among cases with confirmed maternal infection (n/N)	Reference
Rio de Janeiro, Brazil	September 2015–May 2016	Symptoms of infection (rash)	53% (182/345)	RT-PCR	N.A.	N.A.	46% (58/125†)	17
Belem, Pará, Brazil	October 2015–December 2017	Symptoms of infection	44% (134/308)	RT-PCR, serological	N.A.	N.A.	2% (2/101‡)	23
Manaus, Brazil	November 2015–December 2016	Symptoms of infection	42% (322/762)	RT-PCR	N.A.	N.A.	17% (56/322)	66
Pernambuco, Brazil	December 2015–June 2017	Symptoms of infection (rash)	40% (277/694)	RT-PCR, serological	N.A.	N.A.	24.3% (45/185¶)	25
French Guiana	January 2016– July 2016	Confirmed infection	100% (300/300)	RT-PCR, serological	26% (76/291)	RT-PCR or serological	14% (42/291§)	18
São José do Rio Preto, São Paulo, Brazil	February 2016– October 2016	Symptoms of infection	26% (54/216)	RT-PCR	35% (18/51)	RT-PCR and serological	28% (15/54)	20
French territories in the Americas	March 2016– November 2016	Symptoms and confirmed infection	100% (546/546)	RT-PCR	N.A.	N.A.	7% (39/546)	19
Jundiaí, São Paulo, Brazil	March 2016–August 2017	Attendance at high-risk pregnancy clinic	8% (44/574)	RT-PCR	5% (19/409)	RT-PCR	23% (10/44)	24
La Virginia and Dosquebradas, Colombia	2016	Confirmed infection	100% (86/86)	RT-PCR	N.A.	N.A.	23% (20/86)	21

*The range of clinical features considered to be adverse outcomes varies between studies.

†n=125/182 ZIKV-positive pregnant individuals were followedand had known pregnancy outcomes.

‡n=101/134 ZIKV-positive pregnant individuals were followedand had known pregnancy outcomes.

§n=291/300 ZIKV-positive pregnant individuals were followed and had known pregnancy outcomes.

¶n=185/503 children who were able to be evaluated for at least one adverse outcome.

N.A., not available; RT-PCR, reverse transcription polymerase chain reaction; ZIKV, Zika virus.

### 4. There should be a recognisable latent or early symptomatic stage

While highly sensitive and specific laboratory diagnostic assays will improve the identification of maternal ZIKV infection before the onset of severe fetal anomalies, there is currently no available antiviral for impeding vertical transmission of ZIKV in humans^27^ or methods to accurately identify those fetuses who may be ZIKV-exposed but uninfected. Another complicating factor in recognising pregnancies at risk is the large fraction of asymptomatic ZIKV infections,^28^ which are reported to have a similar likelihood of adverse birth outcomes as symptomatic infections.^29^

### 5. The test should be acceptable to the population

While further research on attitudes of expectant families in relation to ZIKV screening is needed, non-invasive prenatal screening tests for congenital infections are generally well accepted.^30 31^ A study in Malaysia found that 81.8% of pregnant individuals who attended antenatal care were willing to be tested for ZIKV, with the lowest percentage found in those in the third trimester.^32^ Effective antenatal education on the various benefits and risks of a ZIKV screening programme may further increase public acceptance.

### 6. There should be an agreed policy on whom to treat as patients

Due to the potential for vertical transmission of ZIKV infection during pregnancy, both the pregnant person and offspring would be considered as patients. While evaluations, clinical decision making and treatment after birth are mainly focused on the offspring, it is crucial to recognise the unique vulnerabilities of parents of CZS patients, such as the increased risk of developing anxiety and depression.^33^

### 7. There should be an accepted treatment for patients with recognised disease

At present, there are no available vaccines to prevent maternal infections or approved antiviral treatments to limit ZIKV infection or block vertical transmission and acute clinical care for ZIKV infection in pregnancy is mainly focused on treating maternal symptoms with analgesics and antipyretics.^34^ In the absence of specific antiviral treatments, the screening programme would aim to counsel expectant parents on CZS-related risks and clinical decisions including, in certain settings, the option of elective termination of the pregnancy, as is the case for cytomegalovirus,^35 36^ and to identify prenatally ZIKV-exposed children for further monitoring and/or treatment for CZS.

Of note, access to unrestricted legal abortion remains rare in Latin America, with the majority of countries having complete prohibition or restrictive laws. Thus, termination is not an option for many pregnant individuals with ZIKV infection, even if microcephaly and/or brain abnormality are detected at ultrasound examination or ZIKV is detected early in the pregnancy. However, there are other interventions that can be offered to pregnant persons and their families, such as supportive measures to mitigate the emotional impact of the potential adverse outcomes.^37^

For newborns with CZS, anticipatory guidance to caregivers and early referrals to appropriate specialists and support groups is crucial. Although confirmatory research is ongoing, early intervention programmes addressing CZS are well accepted and expected to result in an overall improved functional performance.^38^

### 8. Facilities for diagnosis and treatment should be available

In the absence of authorised point-of-care rapid tests,^39^ ZIKV diagnostics rely on expensive, high-complexity and time-intensive diagnostic RT-PCR and serological assays requiring skilled technicians, laboratory facilities and specialised equipment. Further, adequate follow-up care for ZIKV-exposed pregnancies and tertiary care for children with severe manifestations of CZS (eg, severe microcephaly) requires significant on-going financial commitment from public health systems.

### 9. The cost of case finding should be economically balanced in relation to possible expenditure on medical care as a whole

The cost of a prenatal infection screening programme depends on the annual number of pregnancies, whereas the preventable costs depend on the maternal infection rate. Thus, if the screening costs are less than the preventable costs, then a screening programme will be cost-effective and should be adopted.^40^ This is likely to be the case during a ZIKV outbreak, when local maternal infection rates would be increased and the positive predictive value (PPV) of testing would be at its optimal.

### 10. Case finding should be a continuing process and not as a once-and-for-all project

Continuing examinations allow a programme to become more efficient and economical, and regular offers of examination are likely to gradually cover more and more of the population at risk. In addition to or in lieu of laboratory testing, continued monitoring and case finding of at-risk pregnant individuals can be done using ultrasound. Although microcephaly was initially described as the hallmark feature of *in utero* exposure to ZIKV infection and presentation of CZS at birth,^41^ it is now well accepted that newborns can have CZS in the absence of microcephaly.^42 43^ Notably, head circumference (HC) may be increased in some cases due to severe ventriculomegaly.^44^

Given reports of late-onset ZIKV-associated anomalies among children with a normal HC at birth,^45^ it is recommended that healthcare providers remain vigilant for possible sequelae of congenital infection^46^ even in the absence of typical phenotypic features of CZS.^47^ The US Centers for Disease Control and Prevention recommends laboratory testing of infants with clinical features of CZS regardless of maternal laboratory results, as well as ZIKV-exposed infants without CZS features.^46^ While the specific clinical evaluation and management of CZS depends on the ZIKV status of the infant and/or mother, it is recommended that a cranial ultrasound, as well as audiology and ophthalmology assessments are performed by 1 month of age.^46^ Follow-up for 6 months to 1 year is also advised to assess for the development of any later onset clinical manifestations that could be associated with CZS (eg, epilepsy, developmental delays or delay in head growth)^46^ and to facilitate prompt referral to appropriate specialists, and early intervention programmes if required.^46^

## Prenatal ZIKV screening in practice: benefit versus harm

A major challenge in implementing a screening programmes is balancing the benefits with the harms of screening.^1^ The benefit of prenatal screening for ZIKV during an epidemic would be implementation of early interventions that can better support families by providing parents with information to help make informed choices, as well as providing newborns with specialist care. There are also societal benefits of an antenatal screening programme such as the potential costs saved by preventing long-term disabilities. A screening programme could also drive positive changes in a health system that provides better overall support for families who care for children with congenital disabilities, as well as potentially driving research for improved diagnostic testing and antiviral treatments. Additional benefits of prenatal screening for congenital infections include an opportunity to educate seronegative individuals about behaviours that increase risk of transmission.^48^ For example, a study in France showed improved hygiene education for expectant mothers and their partners reduced cytomegalovirus infection rates during the pregnancy.^49^

As there will always be false positives and false negatives, a screening programme may introduce unintended harms.^1^ Whereas false-negative tests (ie, low sensitivity) will lead to missed opportunities for further evaluation and delayed detection of CZS,^50^ false-positive tests (ie, low specificity) have the potential to lead to adverse outcomes including potentially unwarranted pregnancy terminations. A screening test’s PPV and negative predictive value (NPV) (ie, the probability that subjects with a positive or negative screening result truly do or do not have the condition, respectively) depend not only on the sensitivity and specificity of the screening test but also on the prevalence of the condition in the population. In a population with low ZIKV circulation, screening would result in a high proportion of false positives requiring further invasive confirmatory testing, which brings additional risks to the fetus and mother, as well as the potential psychosocial repercussions that need to be considered.^51^ In addition to the impact on the individual/family, the economic costs of screening to a country’s health system is also important, especially if the majority of individuals being screened do not have the condition.

Another factor that complicates the benefit–harm analysis is the current uncertainty in the estimated risk of adverse outcomes following detection of a ZIKV infection during pregnancy, which may challenge the clinical decision-making process and be a source of significant anxiety to expectant parents. Given that only limited quantities of new data are likely to become accessible before the next reemergence of ZIKV, large-scale individual participant data meta-analyses of existing cohort studies, such as those proposed by the Zika Brazilian Cohorts Consortium,^52^ the European Commission Zika Consortia Vertical Transmission Study Group^53^ and the Zika Virus Individual Participant Data Consortiun,^37 54^ will be essential for standardising exposure and outcome definitions and improving understanding of the full spectrum of CZS outcomes including rare outcomes, the precision of risk estimates, and the sources of heterogeneity in the risk estimates. In addition, efforts to link the longitudinal electronic health records (eg, hospitalisations and deaths) of children in ZIKV-affected regions, such as through the Centro de Integração de Dados e Conhecimentos para Saúde Birth Cohort in Brazil,^55^ will yield valuable insights regarding the long-term prognosis of children with CZS.

Ultimately, the decision to implement a prenatal ZIKV screening programme is highly dependent on the context of a country’s health system, resources available and guiding ethical principles. Screening criteria that incorporate the values and priorities of a country and its population may help balance the benefits and harms of a screening programme. Anticipated benefits and harms can be tested using pilot projects, which will also generate important data about cost-effectiveness and efficiency. Additional research that more precisely defines risk estimates of adverse fetal outcomes or leads to the development of lower cost and/or more accurate diagnostics or of novel therapeutics will also likely tip the scale.

## Potential strategies for prenatal ZIKV screening during an outbreak

**Figure 1:**
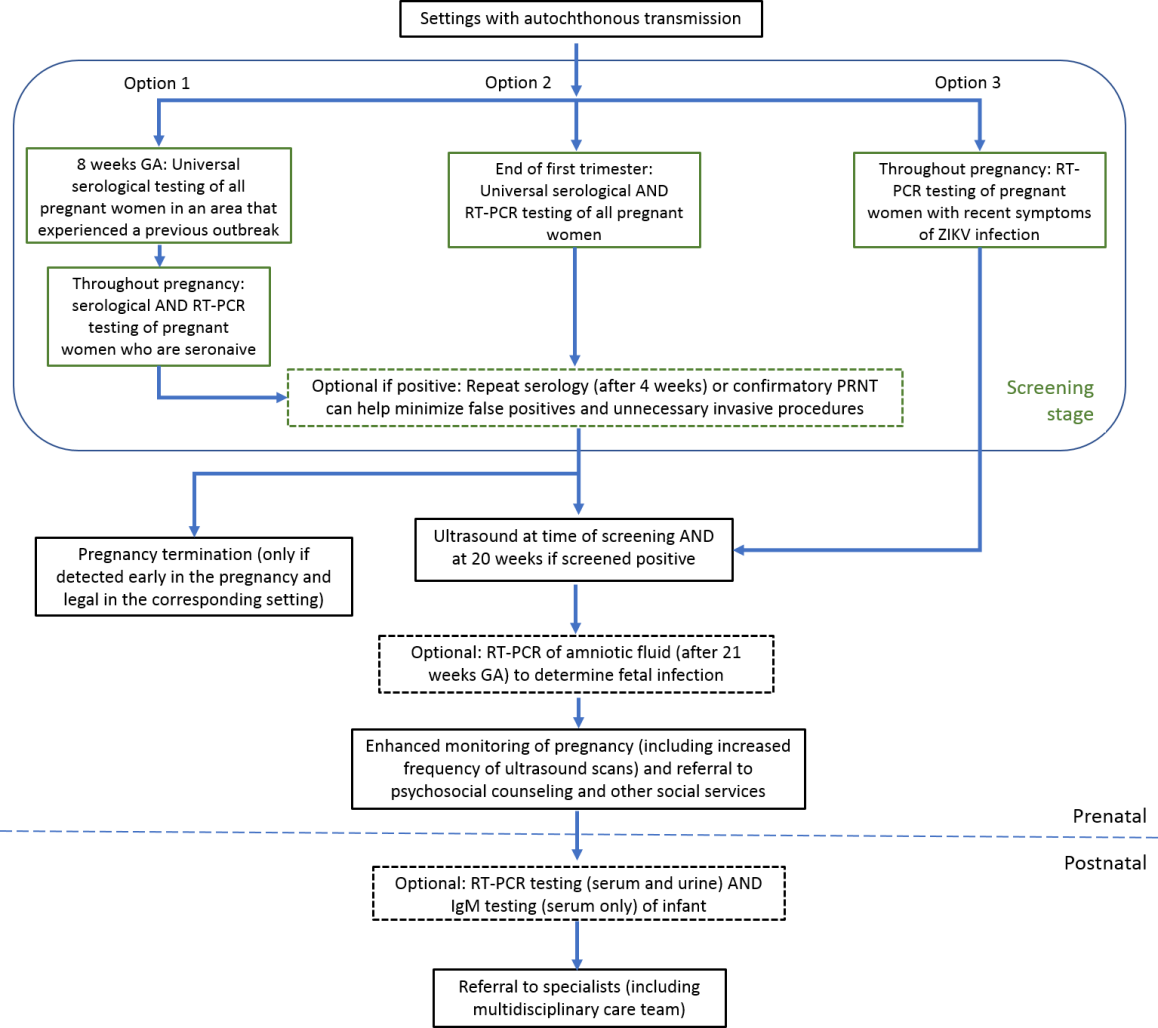
Potential ZIKV screening strategies during pregnancy. This flow chart illustrates the progression for different CZS screening strategies during pregnancy with options for increased confirmatory testing depending on resources available.^65^ CZS, Congenital Zika Syndrome; GA, gestational age; IgM, immunoglobulin M; PRNT, plaque reduction neutralisation test; RT-PCR, reverse transcription-polymerase chain reaction; ZIKV, Zika virus.

To help illustrate population-based prenatal screening for ZIKV, here, we present a general flow-diagram with recommended components that can be further elaborated to fit each settings’ needs and available resources. In settings with a history of recent ZIKV circulation and a high seroprevalence, a universal screening strategy based on serological testing at the first antenatal visit could be adopted to identify seronaive individuals who may be at heightened risk of ZIKV infections and would benefit from repeated testing during pregnancy (figure 1; option 1). The chief limitations of this approach are cross-reactivity with DENV and other flaviviruses^56^ as well as false reassurance and reduced preventive behaviours and prenatal monitoring.^15^ An alternative approach for universal screening would be to test all pregnant individuals near the end of the first trimester using a combination of serological and molecular assays to detect recent and acute infections (option 2). While testing could be repeated during the second or third trimester, current evidence suggests maternal infection during the first trimester is associated with the highest risk of CZS and vertical transmission,^19 39 57^ and the option of elective pregnancy termination remains available in some, but not all, countries. Lastly, ZIKV screening via molecular testing could be targeted to pregnant individuals who present with symptoms consistent with acute ZIKV infection (option 3). Although targeting only symptomatic individuals can help reduce operating costs, more than 60% of ZIKV infections that are asymptomatic may be missed.^28^ A potential strategy that can be added to option 3 to reduce cost and to cover asymptomatic infections, is to pool asymptomatic samples before RT-PCR with workup of individual samples only if pooled test results are positive. This has been used with sufficient diagnostic accuracy for SARS-CoV-2 with pool sizes up to 30 samples.^58^

Modification to the diagram may be warranted in settings with mostly travel-related infections. ZIKV seroprevalence varies hugely among traveller populations compared with resident populations living in areas with active transmission (<2% in travellers vs up to 60% in the local population,^16^ due to differing risks of exposure as well as access to and affordability of preventive measures.^59^ For settings with mostly travel-related transmission, a routine questionnaire, which is recommended by the American College of Obstetricians and Gynecologists to identify possible exposure before considering the need for testing,^60^ can be applied first to identify those at risk before initiating any of the options presented.

Following a positive screening result, an ultrasound should be obtained as evidence suggests a normal HC to femur length ratio in fetuses without microcephaly is associated with an 87% NPV for the postnatal detection of congenital ZIKV-associated injuries.^61^ Further laboratory testing to determine offspring infection status can be performed either before (via amniocentesis, which may introduce additional risks to the pregnancy and for which data regarding PPV and NPV are unknown^62^) or as soon as possible after birth (using serum and/ or urine).^24 62^ In addition to standard evaluations, all infants with known prenatal exposure to ZIKV infection should also receive a head ultrasound, an opthalmological examination, and a hearing examination. Lastly, infants with laboratory or clinical evidence of CZS should have referrals to early intervention service programmes and family support services in addition to clinical consultations regarding child development, infectious disease, neurology, and other specialities as indicated based on clinical findings.^62^

## Timing for implementing a prenatal ZIKV screening programme

A crucial prerequisite to the effective implementation of a screening programme is surveillance. By systematically monitoring the incidence of infections and associated disease, such as congenital anomalies, surveillance helps to inform the timing and type of screening programme adopted by providing information on the rate of community-wide transmission. In addition, long-term surveillance may also yield information on the likely proportion of the pregnant population with pre-existing protective immunity to ZIKV. Although there is limited evidence on the duration of neutralising antibodies following a ZIKV infection,^63^ it is plausible that, in populations with a high seroprevalence following an outbreak (eg, 63% in Salvador, Brazil),^64^ there may be a lower maternal infection rate in future epidemics. Thus, given the synergistic utility of screening and surveillance, resuming or establishing the latter may be a necessary preamble to effective implementation of a screening programme.

## Conclusion

Designing a screening programme for ZIKV during pregnancy is a complex process that requires careful consideration. Maternal infection is often asymptomatic or consists of only mild, non-specific symptoms and vertical transmission rates are variable but have potentially life-altering consequences. Normally, screening provides medical benefits from early treatment. However, while early intervention allows for anticipatory support of newborns and families affected by CZS, there are no treatments currently available to prevent the vertical transmission of ZIKV or to minimise the risk of potential congenital anomalies, and termination of pregnancy is not always a legal option. This initial assessment of the feasibility and utility of a prenatal ZIKV screening programme using the Wilson and Jungner criteria highlights affirmative factors such as acceptability and potential positive impact of such a programme, even in the absence of curative treatment and accurate diagnostics. In addition, consideration of the timing, as well as the overall benefits and harms of implementing a prenatal ZIKV screening programme are necessary foundations for more context-dependent decision making. Moving forward, research should be directed into the natural history of CZS, especially the long-term prognosis of ZIKV-exposed children who present with defects before or at birth and those who present asymptomatically at birth but develop clinical manifestations later on. The development of screening tests with enhanced precision and decreased costs and the introduction of efficacious, safe treatment will further improve the benefit-to-risk balance.

## Data Availability

All data are based on published articles and any relevant abstracted data are available in the article.
